# Synthesis, Characterization and Drug Loading of Multiresponsive *p*[NIPAm-*co*-PEGMA] (core)/*p*[NIPAm-*co*-AAc] (Shell) Nanogels with Monodisperse Size Distributions

**DOI:** 10.3390/polym10030309

**Published:** 2018-03-13

**Authors:** Rajesh Raju, Sulalit Bandyopadhyay, Anuvansh Sharma, Susana Villa Gonzalez, Per Henning Carlsen, Odd Reidar Gautun, Wilhelm Robert Glomm

**Affiliations:** 1 Department of Chemistry, Norwegian University of Science and Technology (NTNU), N-7491 Trondheim, Norway; susana.v.gonzalez@ntnu.no (S.V.G.); phjcarlsen@gmail.com (P.H.C.); 2 Ugelstad Laboratory, Department of Chemical Engineering, Norwegian University of Science and Technology (NTNU), N-7491 Trondheim, Norway; anuvansh.sharma@ntnu.no; 3 Polymer Particles and Surface Chemistry Research Group, SINTEF Industry, N-7465 Trondheim, Norway

**Keywords:** *p*NIPAm, crosslinker, monodispersity, VPTT, core-shell, L-DOPA

## Abstract

We report the synthesis and properties of temperature- and pH-responsive *p*([NIPAm-*co*-PEGMA] (core)/[NIPAm-*co*-AAc] (shell)) nanogels with narrow size distributions, tunable sizes and increased drug loading efficiencies. The core-shell nanogels were synthesized using an optimized two-stage seeded polymerization methodology. The core-shell nanogels show a narrow size distribution and controllable physico-chemical properties. The hydrodynamic sizes, charge distributions, temperature-induced volume phase transition behaviors, pH-responsive behaviors and drug loading capabilities of the core-shell nanogels were investigated using transmission electron microscopy, zeta potential measurements, dynamic light scattering and UV-Vis spectroscopy. The size of the core-shell nanogels was controlled by polymerizing NIPAm with crosslinker poly(ethylene glycol) dimethacrylate (PEGDMA) of different molecular weights (*M_n_*-200, 400, 550 and 750 g/mol) during the core synthesis. It was found that the swelling/deswelling kinetics of the nanogels was sharp and reversible; with its volume phase transition temperature in the range of 40–42 °C. Furthermore, the nanogels loaded with *l*-3,4-dihydroxyphenylalanine (L-DOPA), using a modified breathing-in mechanism, showed high loading and encapsulation efficiencies, providing potential possibilities of such nanogels for biomedical applications.

## 1. Introduction

Stimuli-responsive polymers belong to a class of smart materials that have the ability to respond to a variety of external stimuli; like pH [1], temperature [2], mechanical force [3], the presence of various molecules [4] and electric/magnetic fields [5,6,7]. Core-shell polymeric materials in the form of nanoparticles [8,9], nanocomposites [10,11] and nanofibers [12,13] have attracted wide attention in the field of drug delivery. Response to stimuli, distribution of the loaded drug and other physico-chemical properties of such nanomaterials determine their end-uses. 

Among the stimuli-responsive polymers, *N*-isopropylacrylamide (NIPAm)-based gels that can deliver potential drug molecules under the influence of temperature, pH or ionic strength are of paramount importance [1,14,15,16,17,18,19]. NIPAm-based polymers (for instance, *p*NIPAm) undergo an entropy-driven swelling-collapse behavior above 32 °C, the lower critical solution temperature (LCST) [20]. The isopropyl groups in the *p*NIPAm side chains play a major role in this temperature-dependent phase separation [2,21]. NIPAm-based gels can be modified to have higher or lower transition temperature by incorporating various comonomers and crosslinkers [22,23]. Copolymerizing NIPAm with ionic monomers and comonomers results in temperature-sensitive nanogels with an increased volume phase transition temperature (VPTT) [21,24]. Nolan et al. have reported that by incorporating polyethylene glycol (PEG) as a crosslinker, there is a considerable increase in the phase transition temperature and breadth of the phase transition [25]. Furthermore, incorporation of hydrophilic PEG chains in the nanogels allows tuning of their VPTT and hence control of their swelling-collapse characteristics [26,27]. Grafting PEG chains to the nanogels also affects their swelling behavior, leading to an increased VPTT and broadening of the phase transition [28,29,30].

To be used as hydrophilic drug carriers, *p*NIPAm nanogels should demonstrate a VPTT higher than 37 °C and have active sites for higher drug loading capacities [31,32]. In order to fulfil these criteria, the *p*NIPAm homopolymer requires some modifications. This is because the homopolymer lacks a sufficient drug loading capacity to a high enough concentration owing to the absence of a three-dimensional (3D) network [33]. Furthermore, its temperature sensitivity is around 30–34 °C, which is lower than the normal temperature of the human body [34]. This will lead to a premature release of drug from the hydrogel capsules due to the shrinkage of hydrogels during blood circulation in normal body fluids of 37 °C, widely distributing in vivo and giving poor therapeutic effects [32]. Setbacks related to increasing the NIPAm’s VPTT and hydrophilic drug loading can be resolved by polymerizing NIPAm with ionic comonomer (acrylic acid (AAc)) and hydrophilic crosslinker (poly(ethylene glycol) dimethacrylate (PEGDMA)). However, polymerizing NIPAm with crosslinker PEGDMA will result in nanogels with inadequate sharp transition [25], and fabrication of NIPAm nanogels with a higher AAc concentration can lead to two-step VPTTs [35]. In some cases, strong electrostatic repulsion between the AAC units can even smear out the temperature-induced transition [36].

One way to overcome the above-mentioned shortcomings is by designing NIPAm nanogels with a core-shell architecture; the core comprising NIPAM and PEG and the shell comprising NIPAm and AAc. The resulting nanogels will have increased VPTT and higher hydrophilic drug loading capacity. Moreover, NIPAm nanogels with such a core-shell morphology would illustrate dual responsive properties with respect to both temperature and pH. Separate studies have, so far, investigated the effect of either temperature on NIPAm gels [21,37], or pH [38,39], or both temperature and pH on core-shell gels [40,41,42]. A number of researchers have focused on the synthesis of NIPAm gels with increased VPTT (37–42 °C) for hyperthermal drug release [40,43,44] or synthesis of NIPAm gels with a focus on increasing the drug loading capacity. In contrast, little work has been focused on synthesizing dual responsive (temperature and pH) nanogels with a core-shell morphology, along with tunable VPTT in the range of 37–42 °C for applications in hyperthermal drug release while simultaneously focusing on the gels to have increased drug loading efficiency.

In this article, we report a comprehensive study focusing on the synthesis and properties’ characterization of temperature and pH sensitive core-shell nanogels (*p*[NIPAm-*co*-PEGMA] (core)/*p*[NIPAm-*co*-AAc (shell)]) with VPTT between 40 and 42 °C. The nanogels were synthesized using dispersion polymerization and seeded precipitation polymerization methods and were found to have narrow size distributions. The influence of crosslinker with varying molecular weights in the core of the nanogels was investigated to understand its role in determining the physicochemical properties of the core-shell nanogels. The nanogels showed high loading and encapsulation efficiencies with *l*-3,4-dihydroxyphenylalanine (L-DOPA), a drug for Parkinson’s disease [45].

## 2. Experimental

### 2.1. Materials

*N*-Isopropylacrylamide (NIPAm), poly(ethylene glycol) dimethacrylate (PEGDMA) (average *M_n_* 400, 550 and 750 g/mol), poly(ethylene glycol) methacrylate (PEGMA) (average *M_n_* 360), acrylic acid (AAc), *N*,*N*′-methylenebis(acrylamide) (BIS), ammonium persulfate (APS), sodium dodecyl sulfate (SDS) and 3,4-dihydroxy-l-phenylalanin (L-DOPA) were purchased from Aldrich and used as received. Poly(ethylene glycol) dimethacrylate (PEGDMA) (average *M_n_* 200) was purchased from Polysciences. Uranyl acetate (SPI chemicals) was used as the staining agent. All solutions were prepared using distilled de-ionized water (resistivity ~18.2 µΩ-cm) purified by the Simplicity^®^ Millipore water purification system. TEM (transmission electron microscope) copper carbon grids (300 mesh) were purchased from Electron Microscopy Sciences, Fort Washington. Cellulose dialysis tubing (Sigma-Aldrich, St. Louis, MI, USA) with a molecular weight cut-off (MWCO) of 14 kDa was used both for performing dialysis and purification of the nanogels.

### 2.2. Synthesis of Core Nanogels

*p*[NIPAm-*co*-PEGMA (*M_n_* 360)] nanogels were prepared using PEGDMA (average *M_n_* 200, 400, 550 and 750 g/mol) as a crosslinker using free radical dispersion polymerization. PEGDMA *M_n_* 200 was sparingly soluble in water, whereas the other molecular weights were readily soluble in water. The synthetic procedure was adapted from Leobandung et al. and modified accordingly [46]. Briefly, NIPAm, PEGDMA (200, 400, 550 and 750 g/mol) and PEGMA (*M_n_* 360) were dissolved in deoxygenated water (25 mL). The resulting colorless solution was purged with nitrogen for two hours, and APS (0.005 g, 1 mM) was added to the reaction mixture to initiate polymerization. The amounts of different chemicals used for the synthesis of the core nanogels are given in Table 1. The polymerization reaction was carried out at 82–84 °C (internal reaction mixture temperature) for 1 h under nitrogen atmosphere. During this time, the reaction mixture underwent a phase transition from colorless to a turbid solution, comprising the nanogels. This phenomenon arises due to the fact that the formed nanogels are insoluble at this reaction temperature (well above the VPTT of the synthesized nanogels), whereas the monomers are completely soluble in water.

### 2.3. Synthesis of Core-Shell Nanogels

**Figure 1:**
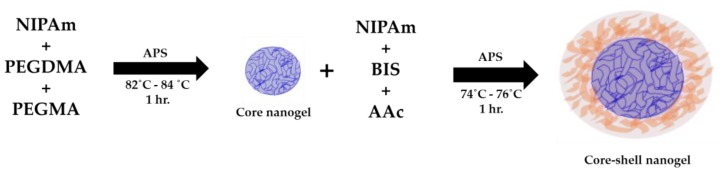
Schematic showing the synthesis of *p*[NIPAm-*co*-PEGMA]/p[NIPAm-co-AAc] core-shell nanogels. BIS, *N*,*N*′-methylenebis(acrylamide); AAc, acrylic acid.

Core-shell nanogels were synthesized via a two-stage seeded polymerization method. This approach has been reported to afford nanogels with core-shell morphology without nucleation of new nanogels [16]. The above synthesized *p*[NIPAm-*co*-PEGMA] core nanogels (10 mL) were dispersed in deoxygenated water (40 mL) containing NIPAm (71%), BIS (15%), AAc (14%) and SDS (5 mM). The resulting turbid reaction mixture was heated to 74–76 °C (internal reaction mixture temperature) under a gentle stream of nitrogen. To this heated mixture, solid APS (0.01 g, 1 mM) was added to initiate the polymerization, and this reaction was then allowed to proceed for 1 h. The reaction mixture was then cooled, transferred into a pre-washed dialysis tube and dialyzed for 12 h. The overall schematic representing the synthesis of core-shell nanogels is depicted in Figure 1.

## 3. Characterization and Measurements

### 3.1. Dynamic Light Scattering and Zeta Potential Measurements

The hydrodynamic size distribution and zeta potential of the nanogels were measured using a Malvern Zetasizer Nano-ZS instrument and the manufacturer’s software. The zeta potential of the nanogels was determined by studying their electrophoretic mobility and then applying the Smoluchowski equation using the manufacturer’s software. All measurements were done in aqueous solutions, and results were averaged over triplicate measurements.

### 3.2. Proton Nuclear Magnetic Resonance 

^1^H NMR spectra were recorded in a Bruker advance DPX400 instrument. Lyophilized nanogels (2 mg) were suspended in D_2_O (0.8 mL), and the spectra were recorded with 128 scans at 25 °C. The reference peak was locked at 4.80 for D_2_O. Chemical shifts (δ) were reported in ppm.

### 3.3. Ultraviolet-Visible Spectroscopy Measurements

UV–Vis spectra were acquired with a UV-2401PC (Shimadzu) spectrophotometer. The spectra were collected over the spectral range from 200–800 nm. The wavelength range was scanned at an interval of 0.5 nm using the instrument’s own software. The results from the spectrophotometer were used in terms of absorbance and analyzed using UVprobe2.10. The absorbance peak for L-DOPA was noted at a wavelength of 287 nm.

### 3.4. Transmission Electron Microscope

The morphology of the nanogels was examined with JEOL TEM-1011. Uranyl acetate was used as the staining agent. In a typical experiment, lyophilized nanogels were suspended in aqueous solution (1 mg/5 mL) and equilibrated at room temperature for 5 h. Prior to imaging, the nanogels were sonicated for 1 min for better dispersion. Uranyl acetate solution (1 mL, 4%) was added to a solution containing nanogels (1 mL) and allowed to equilibrate at room temperature for 15 min. A drop of the resulting suspension was added on the surface of a 300 mesh copper carbon grid. The droplet was allowed to stand on the grid for 5 min and carefully dried using a filter paper. The grid was air dried for 24 h and then clamped onto a TEM specimen rod, inserted into the sample chamber and observed at 80 kV.

### 3.5. Drug Loading Studies

Nanogels were loaded with L-DOPA using a modified breathing-in mechanism [47] and put on a shaker for 2 h. After loading, the solution was centrifuged at 14,500 rpm for 15 min using VIVASPIN centrifugal filters (3000 MWCO). The supernatant obtained was adequately diluted, and the concentration of the drug in the supernatant was measured using the UV-Vis spectrophotometer. The absorbance peak was noted at a wavelength of 287 nm. The loading and encapsulation efficiencies were calculated using the following formulae:

$$\mathrm{Loading}\;\mathrm{Efficiency}\;\left(L.E\right)=\frac{\left(\mathrm{Initial}\;\mathrm{Drug}\;\mathrm{Concentration}-\mathrm{Drug}\;\mathrm{Concentration}\;\mathrm{after}\;\mathrm{loading}\right)}{\mathrm{Initial}\;\mathrm{Drug}\;\mathrm{Concentration}}\times 100$$

$$\mathrm{Encapsulation}\;\mathrm{Efficiency}\;\left(E.E\right)=\;\frac{\mathrm{Loading}\;\mathrm{Efficiency}\times \mathrm{Initial}\;\mathrm{Drug}\;\mathrm{Concentration}\left(\frac{\mu g}{\mathrm{mL}}\right)}{\mathrm{Initial}\;\mathrm{Hydrogel}\;\mathrm{Concentration}\left(\frac{\mathrm{mg}}{\mathrm{mL}}\right)\times 100}$$

## 4. Results and Discussion

**Figure 2:**
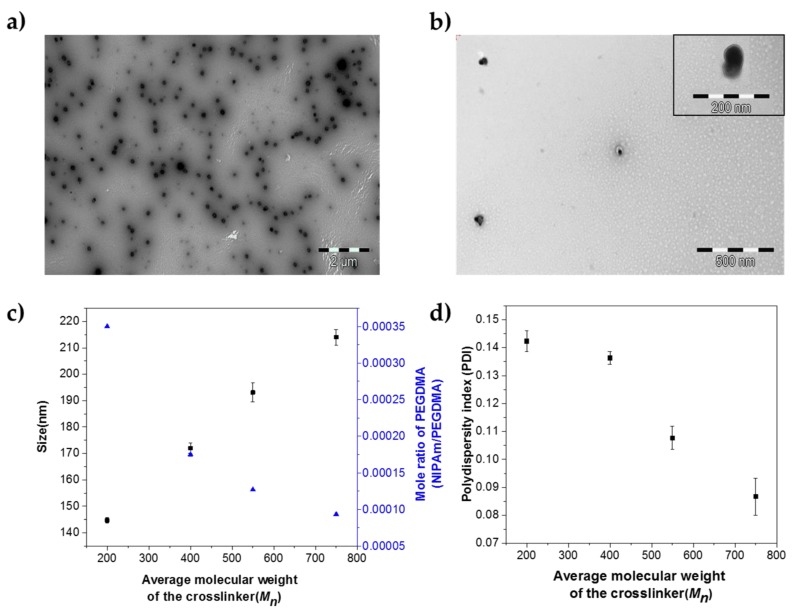
(**a**) Representative TEM images of: (**a**) *p*[NIPAm-*co*-PEGMA] core nanogels; (**b**) *p*[NIPAm-*co*-PEGMA] (core)/*p*[NIPAm-*co*-AAc (shell)] nanogels with uranyl acetate staining. (**c**) Variation in hydrodynamic sizes of the nanogels (squares) and variation in mole ratio of the crosslinker (triangles), as a function of the average molecular weight of the crosslinker (*M_n_*) at 25 °C. The error bar represents the standard deviation from triplicate measurements. (**d**) Variation in polydispersity indices (PDIs) as a function of the average molecular weight of the crosslinker at 25 °C. Error bars represent the standard deviation of triplicate measurements.

The core (*p*[NIPAm-*co*-PEGMA]) nanogels presented here were synthesized using dispersion polymerization at 82–84 °C in water. This is a convenient method for producing spherical nanogels with controlled size and low polydispersity, as the precursors are soluble in the medium, but not the resulting polymers [46]. PEGMA and NIPAM are responsible for maintaining the hydrophilicity and hydrophobicity balance of the core nanogels, respectively. The shell (*p*[NIPAm-*co*-AAc]) was synthesized using seeded precipitation polymerization at 74–76 °C in aqueous media. At this temperature, the core nanogels are in a collapsed state and act as nuclei for shell formation. The collapsed core nanogels capture the growing oligomers, which are also hydrophobic in nature, resulting in preferentially controlled growth of the shell [16,19]. Representative TEM images of uranyl acetate stained core and core-shell nanogels are illustrated in Figure 2a,b, respectively. The ^1^H NMR spectrum of the core-shell nanogels depicts that polymerization has proceeded onwards from its precursor NIPAm to afford *p*NIPAm (Figure S1, Supporting Information). The results of these polymerization methods of core and core-shell nanogels can be seen in SEM/TEM images (Figures S2 and S3, Supporting Information).

The core-shell image suggests that the interface between the core and shell is relatively sharp and not interpenetrated. However, it should be noted that particle size could not be measured using this technique because the nanogels have a tendency to flatten and spread on the TEM grid during sample preparation, resulting in polydisperse nanogels [48].

In this work, the thermosensitive NIPAm core nanogels were crosslinked with PEGDMA (200, 400, 550 and 750 g/mol) and copolymerized with PEGMA. Moreover, the shell was grown from the core nanogels using NIPAm, AAc and BIS as the monomer, co-monomer and cross-linker, respectively. Since the synthetic conditions and precursors are similar for all the samples during shell addition, any change in the size and distribution of the nanogels should have originated from the core’s physico-chemical properties. Thus, incorporating PEG chains as crosslinkers in core preparation can be expected to affect the size and overall size distribution of the core-shell nanogels. Herein, we report the synthesis of core-shell nanogels with a narrow size distribution.

Variation in the molecular weight of the crosslinker induces changes in the size of the core-shell nanogels and their size distribution denoted by their polydispersity index (PDI). Nanogels obtained from polymerizing PEGDMA (200, 400, 550 and 750 g/mol) with NIPAm tend to show an increase in their size and a decrease in their PDI; when there is an increase in the molecular weight of the crosslinker (Table 2).

A decrease in the mole ratio of the crosslinker with an increasing molecular weight of the crosslinker results in nanogels with a gradual increase in hydrodynamic size as seen from Figure 2c. The steady increase in the hydrodynamic radius of the core-shell nanogels must emerge from the increase in the size of their respective core. Increasing the PEG chain length from a relatively shorter chain with *M_n_* 200 g/mol to a larger one of *M_n_* 750 g/mol can dramatically affect the size of the nanogels. As the chain length of the crosslinker increases, the inner compartments (pore sizes) formed as a result of polymerization tend to increase; resulting in the overall increase of the hydrodynamic radius of the synthesized core-shell nanogels. The relationship between the crosslinker (PEGDMA) with varying molecular weight and PDI of the nanogels has been illustrated in Figure 2d. As the molecular weight of the crosslinker increases, the PDI of the nanogels synthesized tends to decrease leading to samples with a narrow size distribution (Table 2). The crosslinker PEGDMA plays a pivotal role as a stabilizer in this surfactant free polymerization approach applied here [49].

Crosslinker PEGDMA functions as a steric stabilizer, which can prevent flocculation and aggregation of the particles being formed. Since this method depends on incipient aggregation of the polymerizing species in the early stages of the polymerization, the number of particles is determined by these growing nuclei. The final size and distribution are primarily determined by the amount of monomer used and the ability of the stabilizer to maintain the colloidal stability of the growing particles. Thus, when the molecular weight of the crosslinker (PEGDMA) is increased from 200–750 g/mol, the number of repeating PEG units in its respective crosslinker increases eventually. Hence, PEGDMA (750 g/mol) with higher repeating PEG units is far superior to its counterparts in stabilizing the formed nanogels leading to lower PDI, while PEGDMA (200 g/mol) with lesser PEG units leads to nanogels with higher PDI (Figure 2d).

As the molecular weight of the crosslinker PEGDMA increases from 200–750 g/mol, the balance between the hydrophilic and hydrophobic units within the crosslinker gets altered, leading to a difference in the solubility of the crosslinkers in an aqueous medium. This was evident from the sparingly soluble nature of PEGDMA 200 g/mol in the aqueous medium, while the other crosslinkers (PEGDMA-400, 550 and 750 g/mol) were readily soluble. Since it is a dispersion polymerization process, the difference in solubility of the crosslinker would also affect the incorporation rate and the distribution of the crosslinker in the core nanogels. These, in due course, change the PDI of the resultant nanogels. As the molecular weight of the crosslinker decreases; the degree of stabilization, the incorporation rate and the distribution of the crosslinker tend to form nanogels with increased PDI (Figure 2d).

**Figure 3:**
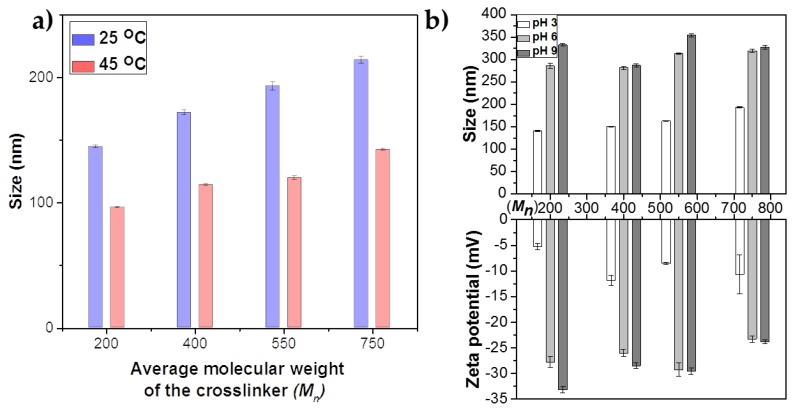
(**a**) Variations in hydrodynamic sizes at 25 and 45 °C. (**b**) Hydrodynamic sizes and zeta potentials of the nanogels as a function of average molecular weight (*M_n_*).

The primary objective of this study was to synthesize core-shell nanogels with higher drug loading capacity, which could be applied for hyperthermal drug release studies (37–42 °C). When the molecular weight of the crosslinker increases, the hydrodynamic sizes of the nanogels gradually increase at 25 °C (Figure 3a). However, when the core-shell nanogels were heated to 45 °C, there is a notable decrease in their hydrodynamic sizes. This decrement in sizes at 45 °C is due to the collapse of the nanogels from their swollen states at 25 °C. At 45 °C, the sizes of the collapsed core-shell nanogels tend to follow the same trend as at 25 °C; an increase in the molecular weight of the crosslinker leads to an increase in the size of the collapsed core-shell nanogels. Consequently, as the mole ratio and the molecular weight of the crosslinker in the nanogels increases, the size of the nanogels tends to increase correspondingly both in swollen and collapsed states.

Figure 3b shows the variation of hydrodynamic sizes and zeta potentials of core-shell nanogels (Mn 200, 400, 550 and 750 g/mol) as a function of pH. An increase in the pH of the individual samples leads to an increase in size and zeta potential of the core-shell nanogels. This effect is due to the presence of polyacrylic acid in and around the shell, which is ionized with increasing pH. The successful incorporation of acrylic acid in the shell was confirmed from potentiometric titrations (see Supporting Information, Figure S5). The thermodynamic model developed by Kost can be used to explain the swelling/deswelling characteristics while changing the pH of the medium [50]. This model considers four sources contributing to the total free energy: (i) nanogels-solvent system; (ii) nanogels-solvent mixing; (iii) deformation of polymer networks; and (iv) osmotic pressure of mobile ions. The nanogels-solvent mixing component is dominated by the poly-AAc segments that undergo dissociation with an increase in pH. Dissociated poly-AAc segments are more hydrophilic than non-dissociated segments, whereby a transition from lower to higher pH causes a drastic decrease in the free energy of mixing. Hydrophobic to hydrophilic transition also explains the consequent swelling of the nanogels. However, this dissociation is affected by the deformation degree of the polymer network, mostly affected by the cross-linking density and the osmotic pressure of OH_−_ and Na^+^ ions. The volume transition that happens in the nanogels at a higher pH is reflective of the fact that the pH is high enough to overcome the osmotic pressure, wherein counter-ion-shielding effects occur within the poly-AAc domains [51]. Once the osmotic pressure is overcome, a synergistic effect of the favorable free energy of nanogel-solvent mixing and decrosslinking of bound poly-AAc segments in the domains causes further swelling with an increase in pH.

The zeta potential not only gives a measure of the surface charge of the nanogels, but also indicates their stability in the solution. A higher surface charge indicates higher stability owing to electrostatic forces. Zeta potential represents the charges contributed by the acrylic acid (AAc) units present in the shell and does not change appreciably as a function of temperature. This is because the zeta potential is only applicable to the nanogels in a semi-quantitative manner as some of the charges are buried and contribute partially to the calculated value [52]. Additionally, there exists no well-defined slipping plane between the nanogel surface and the medium [14]. As the pH of the solution increases, the acrylic acid on the shell ionizes, resulting in a net negative charge. The negative zeta potential values represent the charges contributed by the poly-AAc segments. When the pH increases from 6–9, a higher number of acrylic acid units gets ionized, leading to a higher zeta potential value for the individual nanogels.

**Figure 4:**
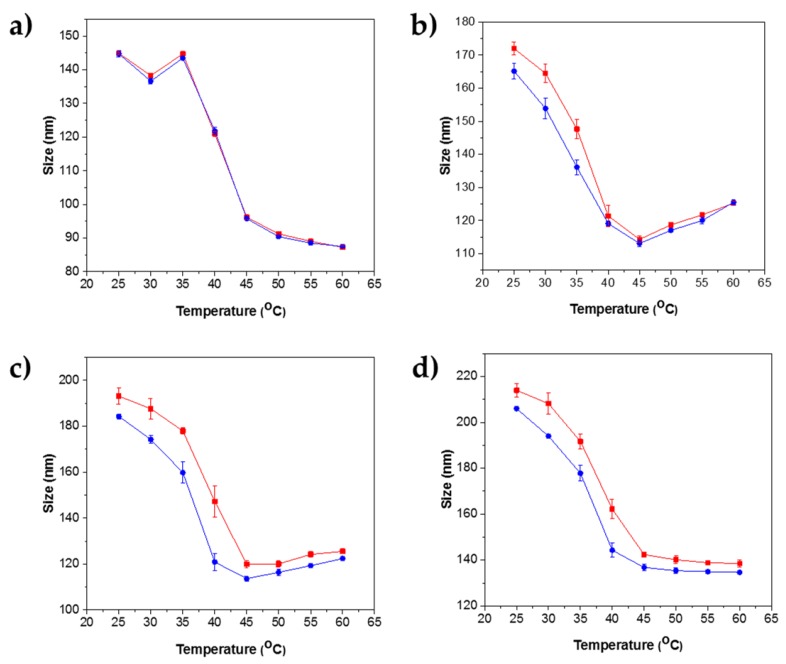
Variations in the hydrodynamic sizes of nanogels as a function of temperature measured using DLS. The red bars indicate heating, and the blue bars indicate cooling. (**a**) Nanogels with PEGDMA (*M_n_* 200 g/mol). (**b**) Nanogels with PEGDMA (*M_n_* 400 g/mol). (**c**) Nanogels with PEGDMA (*M_n_* 550 g/mol). (**d**) Nanogels with PEGDMA (*M_n_*-750 g/mol).

For all four nanogel samples, temperature-induced swelling and shrinking were observed to be completely reversible (Figure 4). Hysteresis is often observed from coil-globule-coil transitions in *p*NIPAm solution due to the formation of a new molten-globule state arising from additional hydrogen bonds in the collapsed state [53]. A key factor involved in this hysteresis was found to be the crosslinker chain length. An increase in hysteresis area was observed with an increase in the chain length, although hysteresis appears to be more pronounced for PEGDMA 550 g/mol than for 750 g/mol. Crosslinker PEGDMA 750 g/mol chains yield larger compartments in the nanogels compared to that of PEGDMA 200 g/mol. For example, compartments formed from PEGDMA 750 g/mol (d1) will have larger interstitial spaces when compared to the compartments formed from PEGDMA 200 g/mol (a1). Thus, when nanogels are heated, the larger compartments allow a reduction in chain entanglement through swelling and collapse cycles. Due to the conformational changes in the chain segments, the compartments may not be restored to their original shapes, and hence, we find greater hysteresis with increasing crosslinker length with PEGDMA 750 g/mol.

Nevertheless, PEGDMA 200 g/mol with small compartments often does not have the required space to allow chain entanglement, and even in a scenario where there is enough space, the conformational changes in the chain segment tend to avoid lone-lone pair repulsion (oxygen in polyethylene groups), which are not energetically favored. Hence, the chain with PEGDMA 200 g/mol tends to reconstruct itself sharply with negligible hysteresis.

In this work, crosslinking PEGDMA of varying molecular weights 200, 400, 550 and 750 g/mol with NIPAm yielded nanogels with a narrow size distribution and modified sizes. PEGDMA being a hydrophilic crosslinker, when incorporated within *p*NIPAm nanogels, changes the hydrophilic balance, leading to an increment in the overall phase transition temperature of the nanogels. The four synthesized nanogel samples exhibit VPTTs ranging from 40–42 °C, calculated from heating and cooling curves depicted in Table 3 using the protocol adapted from Clara Fucinos et al. [54]. The detailed procedure for calculating VPTT is described in Supporting Information (Figure S4), and further reference is made to our previous work [55]. However, we could not observe considerable deviations in the VPTTs of the synthesized samples.

The subtle changes observed in the VPTTs for all four nanogels can be explained by the number of repeating PEO units in PEGDMA. Increasing the PEGDMA chain length from a relatively shorter chain PEGDMA 200 g/mol (approximately five PEO units) to a longer PEGDMA 750 g/mol chain (approximately 17 PEO units) should increase the VPTT of the nanogels. However, in contrast, there are no vast differences in the VPTTs of the samples, indicating that the overall distribution of the PEG unit’s concentration should be approximately identical in all four nanogel samples. The identical concentration of the PEG units in all four samples resulted from varying the concentration of PEGDMA while synthesizing the samples (Table 1), such that the highest MW PEGDMA (750 g/mol) was used at a lower concentration and the lowest MW PEGDMA (200 g/mol) at a higher concentration.

**Figure 5:**
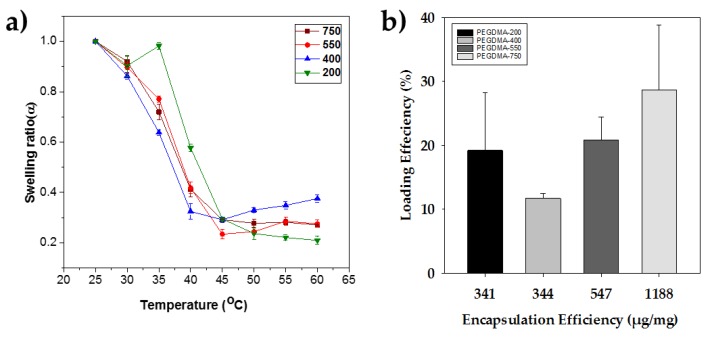
(**a**) Swelling ratios of nanogels as a function of temperature. (**b**) Encapsulation efficiency as a function of loading efficiency for nanogels.

Swelling ratios at different temperatures shown in Figure 5a illustrate the effects of crosslinker chain length on volume phase transition modulation on the nanogels. As the temperature increases, the swelling ratios of all the nanogels decrease. The phase transition temperatures of all the samples were observed to be between 40 and 42 °C, implying that the number of PEO units in nanogels (200, 400, 550 and 750 g/mol) is approximately identical. It is to be observed that the sharpness of the transition is composition dependent. Samples with PEGDMA 400, 550 and 750 g/mol as a crosslinker follow an identical transition; however, nanogels with PEGDMA 200 g/mol in comparison with other samples have a broader transition. Evidently, the broadness of the nanogels with PEGDMA 200 g/mol suggests that there might be an uneven distribution of the crosslinker in the nanogel, either due to a different incorporation rate or phase separation during synthesis.

**Figure 6:**
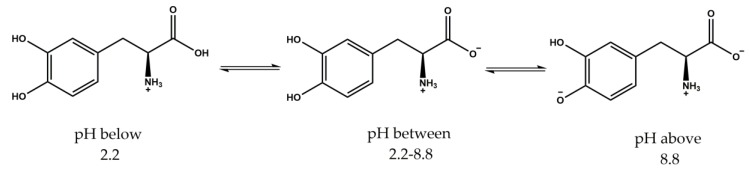
L-DOPA’s plausible ionic structures at different pH values.

The nanogels were loaded with L-DOPA using a modified breathing-in mechanism [52]. In the breathing-in mechanism, the loading efficiency primarily relies on the porosity of the nanogel networks. Hence, we believe that the loading is more dependent on the ease with which the drug can bind to the active sites, and hence, the probable interactions between L-DOPA and the nanogels are discussed here. Although the solubility of L-DOPA will affect retention and eventual release, the effect of partitioning of the drug between the surrounding water and nanogel networks is assumed to be the same for the different systems evaluated here. L-DOPA is a small hydrophilic drug that is soluble in water and that has also been employed as a potential drug in Parkinson’s disease [45,56]. L-DOPA possesses two hydroxyl groups attached to an aromatic ring that can act both as H-bond donors and acceptors, an amine group when in the protonated state, can only act as a hydrogen donor and additionally has a carboxyl group, which in the deprotonated state is an H-bond acceptor [57]. The presence of an amine and a carboxyl group within the molecule in proximity allows L-DOPA to exist in different ionic structures depending on the pH (Figure 6). L-DOPA below pH 2.2 is fully protonated and exists as a +1 cation; whereas above pH 8.8, one hydroxyl group deprotonates to form a net charge of −1; and from pH 2.2–8.8, it exists as a zwitterionic structure in the neutral state [58].

The dispersion containing the nanogels was observed to have a pH 4.3, in which the loaded L-DOPA would exist in the zwitterionic state and could interact with functional groups in and around the nanogels. The free hydroxyl groups in the L-DOPA can form hydrogen bonds with the lone pair of oxygen atoms present in the PEG units of the nanogels and accept hydrogen bonds from the secondary amide groups of NIPAm. L-DOPA in a zwitterionic state with the ammonium ion would interact with the carboxylic or carboxylate ions of AAc in the shell through electrostatic interactions. Therefore, various forces play a prominent role in the encapsulation and loading of L-DOPA in the nanogels.

The loading and encapsulation efficiencies of the nanogels increase with increasing molecular weight of the crosslinker and can be observed from Figure 5b. The increasing efficiency in L-DOPA loading and encapsulation of nanogels with PEGDMA 750 g/mol can be explained by the interaction of L-DOPA within the active sites of the nanogels and the size of the inner compartments present in the nanogels. PEO units in PEG and larger compartment sizes present in the nanogels play a pivotal role in increasing the encapsulation and loading efficiency of the nanogels. Higher molecular weight crosslinker PEGDMA 750 g/mol would have additional repeating PEO units (more active sites) than the lower molecular weight PEGDMA 200 g/mol. As the molecular weight of the crosslinker increases, the length of the crosslinker increases from PEGDMA 750 to PEGDMA 200, resulting in larger compartments with added active sites (PEO units) in comparison to nanogels crosslinked with PEGDMA 200 g/mol. Therefore, nanogels with larger compartments comprising more PEO units would accommodate more L-DOPA molecules through hydrogen bonding. Thus, nanogels with PEGDMA 750 g/mol are found to have a higher loading and encapsulation efficiency than the nanogels with lower molecular weight crosslinkers. To conclude, the decrement in the molecular weight of the crosslinker reduces the size of the compartments, eventually decreasing the loading and encapsulation efficiency, following a trend as shown in Figure 5b.

## 5. Conclusions

*p*NIPAm-based nanogels with core-shell morphology were synthesized, and their morphology was confirmed by TEM studies. By incorporating a hydrophilic and flexible crosslinker such as PEGDMA (200, 400, 550 and 750 g/mol) within nanogels, size modulation of the gels was achieved. DLS studies revealed that the nanogels incorporated with high molecular weight PEGDMA (750 g/mol) yielded a narrow size distribution and the lowest PDI, while the nanogels incorporated with the lowest molecular weight PEGDMA (200 g/mol) showed the highest PDI comparatively. However, incorporation of PEGDMA within the core of the nanogels does not affect the VPTT of the core-shell nanogels appreciably as all the samples show VPTT values in the same range (40–42 °C). Core-shell nanogels with crosslinker PEGDMA (200 and 400 g/mol) showed reversibility in swelling from heating to cooling and vice versa with minimum hysteresis, while samples with crosslinker PEGDMA (550 and 750 g/mol) tend to demonstrate hysteresis. This is attributed to the increase in the chain length of PEGDMA. Swelling ratio studies indicate that the synthesized nanogel samples have sharp phase transition temperatures between 40 and 42 °C. L-DOPA loading and encapsulation studies reveal that the nanogels with higher molecular weight PEGDMA (750 g/mol) demonstrate higher loading and encapsulation efficiencies, while nanogels with low molecular weight PEGDMA (200 g/mol) display the lowest loading and encapsulation efficiencies.

## Figures and Tables

**Table 1:** Chemical compositions used for the preparation of *p*[NIPAm-*co*-PEGMA] core nanogels. APS, ammonium persulfate.

Monomer	Crosslinker (g/mol)	Comonomer	Initiator, (mM)
NIPAm, 84%	PEGDMA(*M_n_*-200), 13%	PEGMA, 3%	APS, 1
NIPAm, 90%	PEGDMA(*M_n_*-400), 7%	PEGMA, 3%	APS, 1
NIPAm, 92%	PEGDMA(*M_n_*-550), 5%	PEGMA, 3%	APS, 1
NIPAm, 93%	PEGDMA(*M_n_*-750), 4%	PEGMA, 3%	APS, 1

Composition in mole%.

**Table 2:** Comparison of hydrodynamic sizes and PDIs of nanogels synthesized using varying PEGDMA at 25 °C.

Average molecular weight(*M_n_*) of PEGDMA (g/mol)	Size (nm)	Polydispersity index (PDI)
200	144 ± 1	0.145
400	172 ± 2	0.135
550	193 ± 4	0.116
750	214 ± 3	0.116

**Table 3:** VPTT values for samples with PEGDMA 200, 400, 550 and 750 (g/mol).

Average molecular Weight (*M_n_*) of PEGDMA (g/mol)	Volume phase transition temperature (°C)
200	41.7 ± 0.1
400	39.9 ± 0.2
550	40.5 ± 0.2
750	41.0 ± 0.3

## Supplementary Materials

The following are available online at http://www.mdpi.com/2073-4360/10/3/309/s1, Figure S1: The ^1^H NMR of the core and the core-shell nanogels in D_2_O, Figure S2: Scanning electron micrographs of *p*[NIPAm-*co*-PEGMA] core particles, Figure S3: Transmission electron micrographs of *p*[NIPAm-*co*-PEGMA] core and *p*[NIPAm-*co*-PEGMA] (core)/*p*[NIPAm-*co*-AAc] (shell) nanogels with uranyl acetate staining, Figure S4: Determination of VPTT, Figure S5: Potentiometric titration curve of *p*[NIPAm-*co*-PEGMA] (core)/*p*[NIPAm-*co*-AAc] (shell) with PEGDMA (200).
