# Supplementary material for: Synthesis, Characterization and Drug Loading of Multiresponsive p[NIPAm-co-PEGMA] (core)/p[NIPAm-co-AAc] (Shell) Nanogels with Monodisperse Size Distributions
Source: Polymers (Basel). 2018 Mar 13;10(3):309. doi: 10.3390/polym10030309 (PMC6414958; doi:10.3390/polym10030309)
Supplement: Supplementary file 1 [file polymers-10-00309-s001.docx]

**Supplementary Information**

Synthesis, Characterization and Drug Loading of Multiresponsive
*p*[NIPAm-*co*-PEGMA](core)/*p*[NIPAm-*co*-AAc] (shell) with Monodisperse Size Distributions

Rajesh Raju^1^*, Sulalit Bandyopadhyay^2^*, Anuvansh Sharma^2^, Susana Villa Gonzalez^1^, Per Henning Carlsen^1^, Odd Reidar Gautun^1^ and Wilhelm Robert Glomm^3^

^1^ Department of Chemistry, Norwegian University of Science and Technology (NTNU), N-7491 Trondheim, Norway.

^2^ Ugelstad Laboratory, Department of Chemical Engineering, Norwegian University of Science and Technology (NTNU), N-7491 Trondheim, Norway.

^3^ Polymer Particles and Surface Chemistry Research Group, SINTEF Industry, N-7465 Trondheim, Norway.

Correspondence: raj.r.j@outlook.com, [sulalit.bandyopadhyay@ntnu.no](mailto:sulalit.bandyopadhyay@ntnu.no), odd.r.gautun@ntnu.no, and wilhelm.glomm@sintef.no

*S1 Proton nuclear magnetic resonance (^1^H NMR)*


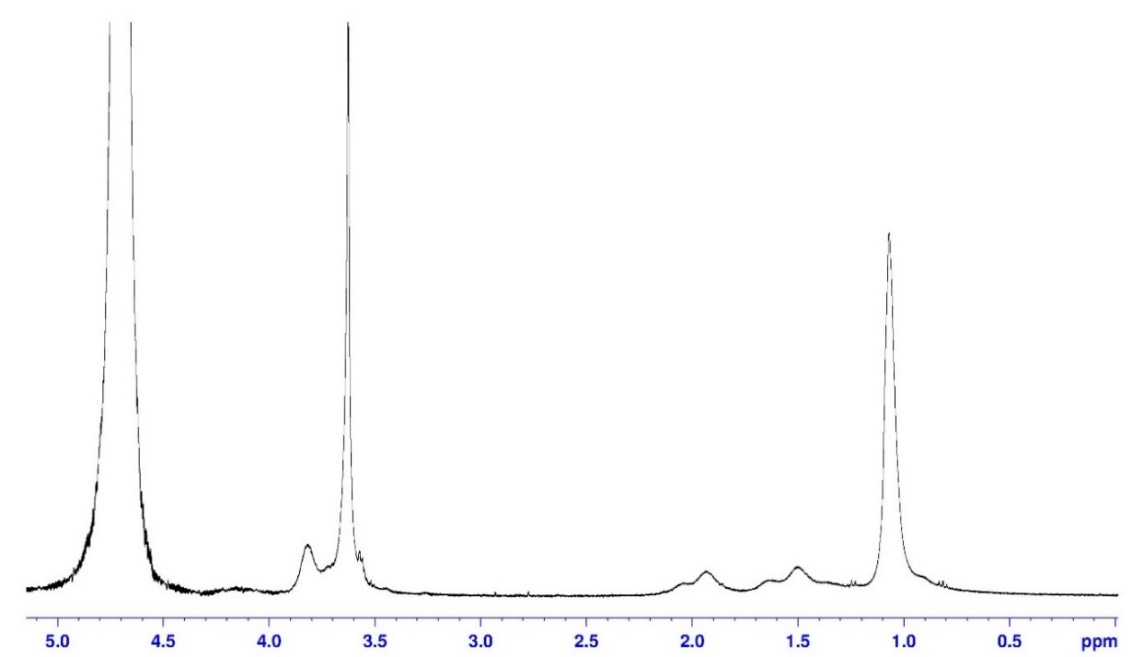
**a)**

**
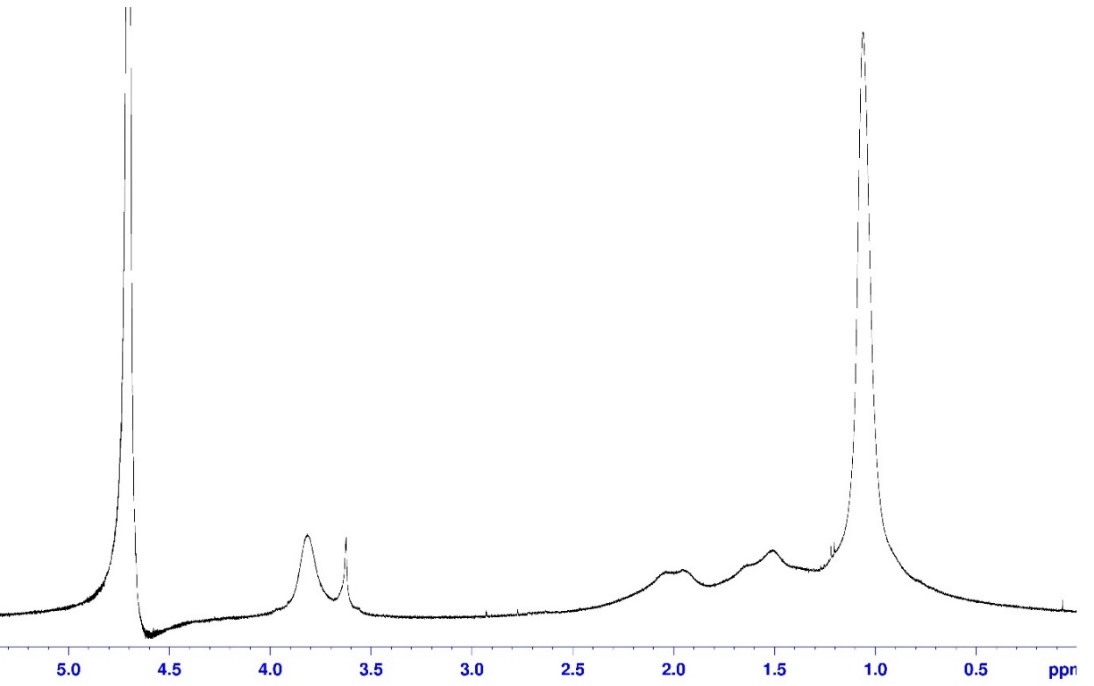
b)**

**Figure S1**. (**a**) ^1^H NMR(D_2_O, 400 MHz) of core nanogels. (**b**) ^1^H NMR(D_2_O, 400 MHz) of
core-shell nanogels.

*S2 SEM*

SEM images were acquired using a JEOL SEM-JSM6480. In a typical experiment, dried nanogels were suspended in Millipore water and sonicated for one minute. A drop of the resulting suspension was spread on the surface of a 200 mesh copper carbon grid (Electron Microscopy Sciences, Fort Washington, PA) and air dried for three hours. The grid was mounted on an aluminum stub with a conductive double-sided carbon tape and coated with Au/Pd using a sputter coater (Polaron coating unit E5100) at 2.5 kV for imaging.

***
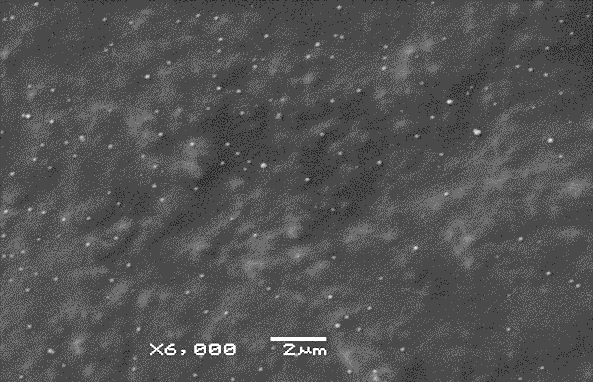
*a**)


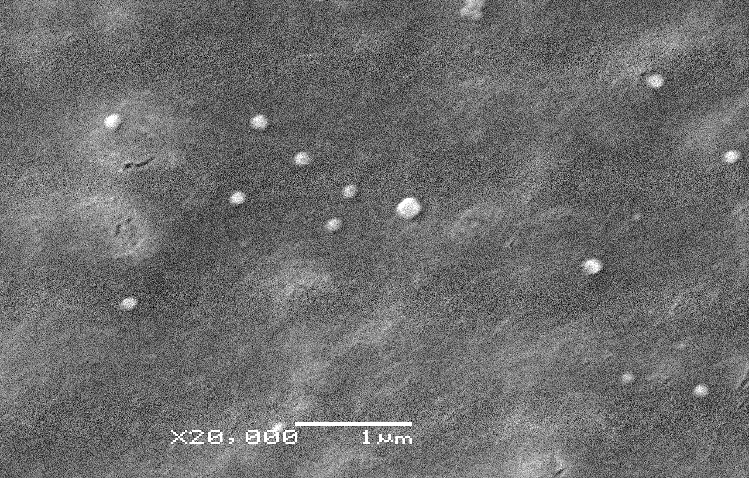
**b**)

**Figure S2.** Scanning electron micrograph (SEM) images of *p*[NIPAm-*co*-PEGMA] core particles.

*S3 TEM*

Core nanogels

*

***a**)



**b**)

Core-shell nanogels


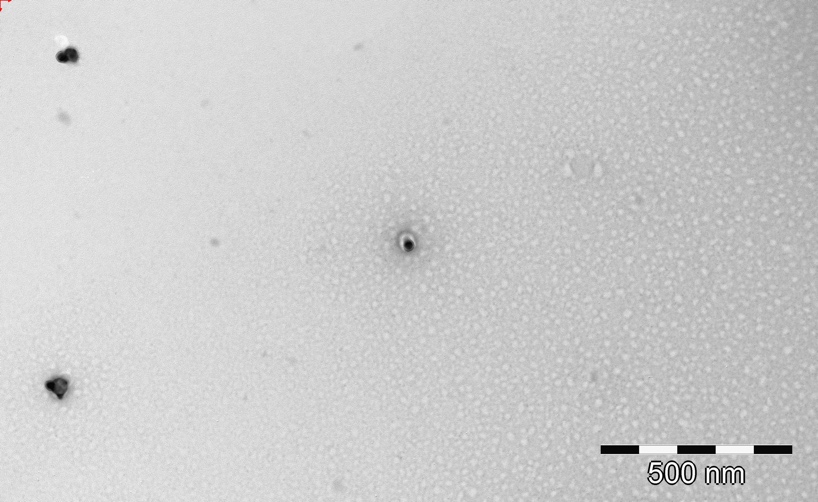
**c**)


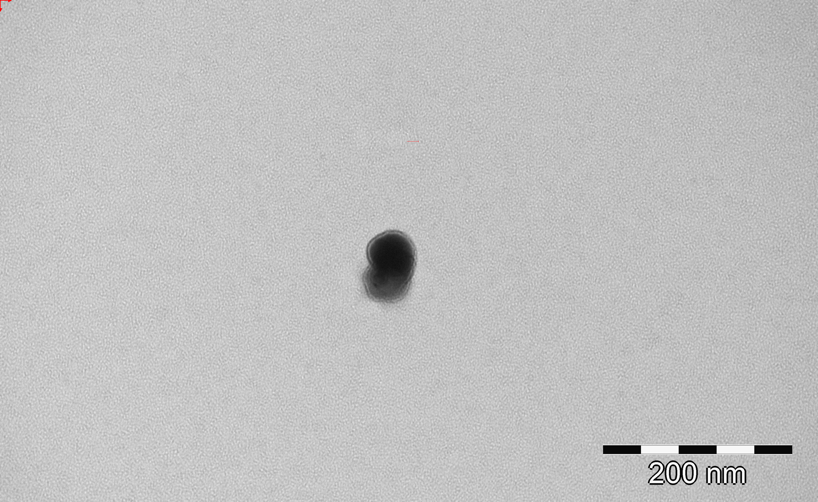


**d**)


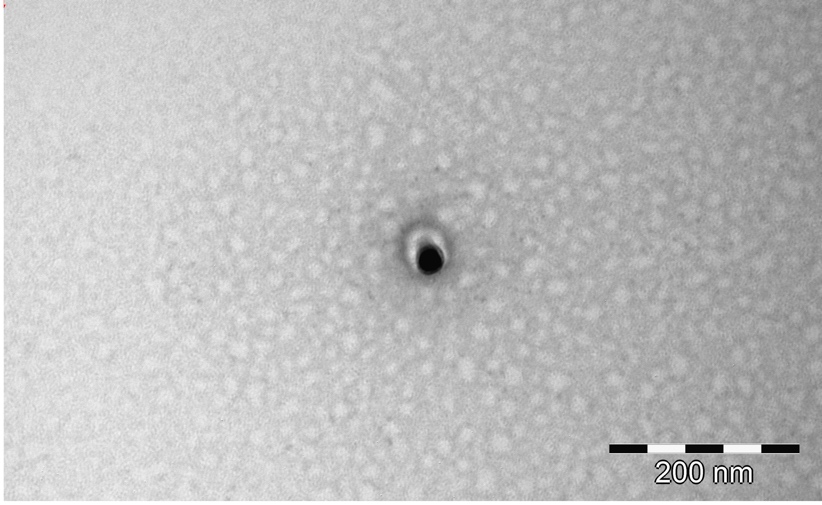
**e**)

**Figure S3.** (**a**,**b**) Transmission electron micrographs of *p*[NIPAm-*co*-PEGMA] core particles with uranyl acetate staining. (**c**,**d**,**e**) Transmission electron micrograph of *p*[NIPAm-*co*-PEGMA]
(core)/*p*[NIPAm-*co*-AAc] (shell) particles with uranyl acetate staining. Core-shell particles with uranyl acetate staining. (c) core-shell particles, (d) fused core-shell particles and (e) core-shell particle.

*S4 Calculations for finding the volume phase transition temperature*

The approach used in the evaluation of collapse temperature involves plotting a spectroscopic parameter along with temperature. The following plot represents the variation of optical density along with absolute temperature.


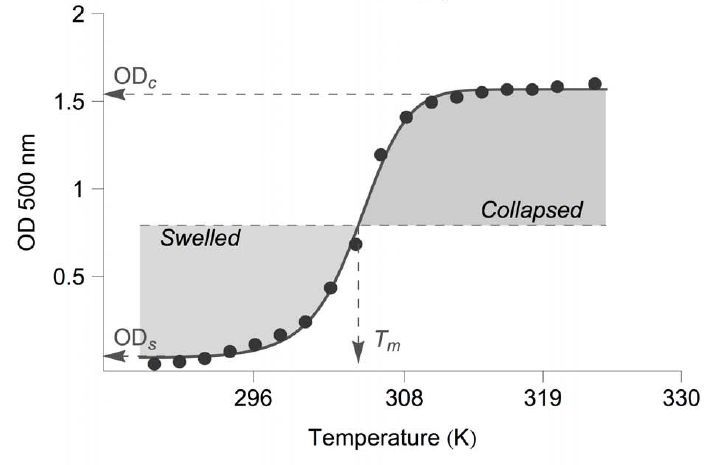


**Figure S4.** VPTT determining graph from Clara Fucinos et al. [1]*.*

The experimental parameter taken into account for our studies is ‘’$\alpha$’’, which is defined as,

$\alpha=\left( \frac{D}{D_{0}} \right)^{3}$ (1)

The nanogel is a mixture of moieties with different sizes, as a result of which the nanogel does not have a distinctive collapse temperature, but varies over a range. At any point on the curve
(Figure S4), the nanogel exists in partially-collapsed states. Therefore, we can assume that at the mean collapse temperature denoted by *T_m_* of the complete nanogel, the collapsed state lies in equilibrium with the swollen state. While the swollen state area lies to the left of *T_m_* on the swelling curve, the collapsed state area lies to the right.

The MATLAB code tries to find out the probable value of the collapse temperature, which is determined by equating the areas under swelling and the collapsed state. Therefore, in the ideal situation, both of these areas should be equal. The area is calculated by integrating the curve, and the method applied here is the “Simpson’s 1/3rd rule” which calculates the area under the curve by integrating the function as follows,

$\int_{a}^{b} f\left( x \right)dx \approx\frac{h}{3} (f\left( x_{0} \right)+2 \sum_{j=1}^{\frac{n}{2}-1} f(x_{2j})+ 4 \sum_{j=1}^{n/2} f\left( x_{2j-1} \right)+f(x_{n}))$ (2)

For the first part of the curve, measured from the lower temperature (*T_a_*), the system integrates between the limits *T_a_* and *T_m_*. Similarly, for the next part of the curve, the limits vary from *T_m_* to the higher temperature limit, i.e., (*T_b_*). SigmaPlot® Version 12.5 is used as a tool to plot the data points and obtain a smooth curve using a sigmoid, five-parameter fit. The fit, shown by Equation (3), provides the users with the values for the five parameters that in turn act as inputs to the MATLAB tm code.

$y=y_{o}+\frac{a}{{(1+e^{-(x-x_{o}/b)} )}^{c}}$ (3)

Then, the code is run, and the user enters these parameters along with the value of relative tolerance, as well as the number of iterations desired by the user. The program calculates the collapse temperature by calculating the relative difference in the areas of the collapsed, as well as the swelled state up to the input tolerance value. The program automatically adjusts the value of the number of iterations, if the desired tolerance is not reached within the iterations provided by the user. At the end of the program run, it generates the curve, thereby confirming with SigmaPlot® curve the dependence, as well as the reliability of the MATLAB code. The point marked with a circle on the curve depicts the collapse temperature of the entire system. It is to be noted that the user is free to enter the temperature in the absolute, as well as in the Celsius scale. The program can be used to study both the heating, as well as the cooling curves.

The choice of the tolerance and the number of iterations has been left to the user. A higher degree of precision has been observed by choosing a lower value of tolerance or a higher number of iterations. For this particular study case, the value of tolerance has been set to 0.001, and the collapse temperature has been calculated by changing the number of iterations. A mean value of all these collapse temperatures is then selected as the overall collapse temperature of the system (separate for heating and cooling).

*S5 Potentiometric titrations*

To verify the incorporation of 0.01 mol% of acrylic acid into *p*[NIPAm-*co*-PEGMA]
(core)/*p*[NIPAm-*co*-AAc] (shell) nanogels, potentiometric titrations were performed with 10 mM NaOH. The nanogels were initially dispersed in water under nitrogen atmosphere with a pH meter, which indicted the dispersion to have a *pK_A_* value of 4.37. This was in agreement with the literature, suggesting that there was effective incorporation of acrylic acid inside the core-shell particles [1]. To avoid possible errors, after the addition of NaOH solution in potentiometric titrations to the dispersion, the mixture was allowed to stir for three minutes and equilibrated for another 10 minutes before the pH was measured.

An example of an obtained potentiometric titration curve is represented in Figure S5 for the *p*[NIPAm-*co*-PEGMA] (core)/*p*[NIPAm-*co*-AAc] (shell).

**Figure S5.** Potentiometric titration curve for *p*[NIPAm-*co*-PEGMA] (core)/*p*[NIPAm-*co*-AAc] (shell) with PEGDMA (200). Potentiometric titrations verify the effective incorporation of acrylic acid in *p*[NIPAm-*co*-PEGMA] (core)/*p*[NIPAm-*co*-AAc] (shell) at a value of at least 95% or greater in all
four samples.

**Reference**

1. Fucinos, C.; Fucinos, P.; Miguez, M.; Katime, I.; Pastrana, L. M.; Rua, M. L. Temperature- and pH-sensitive nanohydrogels of poly(N-Isopropylacrylamide) for food packaging applications: modelling the swelling-collapse behaviour. *PLoS One* **2014**, *9*, e87190, doi:10.1371/journal.pone.0087190
